# Potential protective role of vitamin C in peripheral artery disease: A Mendelian randomization and NHANES analysis

**DOI:** 10.1097/MD.0000000000048220

**Published:** 2026-04-03

**Authors:** Zhiyong Dong, Qingyun Wang, Lisong Hao

**Affiliations:** a Department of Cardiothoracic Surgery, Beijing Shunyi Hospital, Beijing, China.

**Keywords:** Mendelian randomization, micronutrients, NHANES, peripheral artery disease, vitamin C

## Abstract

Although numerous studies have investigated the associations between micronutrients and peripheral artery disease (PAD), most existing evidence is observational and limited by confounding and lack of causal inference. To address these gaps, we combined Mendelian randomization (MR) analysis using genetic data with supportive epidemiologic evidence from National Health and Nutrition Examination Survey (NHANES) to examine the relationships between micronutrient status and PAD risk. This study was conducted in 2 phases. First, we used genome-wide association study summary statistics to assess the causal effects of 15 circulating micronutrients on PAD risk through 2-sample MR. A bidirectional MR was also performed to explore the possibility of a reverse causal effect between PAD and potential candidate micronutrients. In the second phase, we analyzed data from 3 cycles of the NHANES (1999–2004). Logistic regression and restricted cubic spline models were used to examine the association between dietary intake of potential candidate micronutrients and PAD risk, adjusting for relevant covariates. MR analysis showed that higher genetically predicted vitamin C levels were significantly associated with a reduced risk of PAD (inverse-variance-weighted odds ratio (OR) = 0.509, 95% confidence interval (CI): 0.356–0.727; *P* <.001), with no evidence of reverse causality. In the NHANES analysis, lower dietary vitamin C intake was independently and nonlinearly associated with higher PAD risk (overall prevalence 7.9%), showing an L-shaped relationship with a threshold at 225.82 mg/day. Compared to the lowest quartile, PAD risk was lower in the second quartile (adjusted OR = 0.79; 95% CI: 0.64–0.98) and third quartile (adjusted OR = 0.94; 95% CI: 0.63–1.38). No significant interactions were found in the subgroup analysis. Both genetic evidence from MR and supportive observational evidence from NHANES suggest that vitamin C may play a protective role in the development of PAD.

## 1. Introduction

Peripheral artery disease (PAD) is a vascular condition characterized by the narrowing or blockage of arteries that supply blood to parts of the body other than the heart and brain, primarily affecting the legs.^[1]^ The fundamental pathological basis of this disease is atherosclerosis,^[2]^ a process where plaque – composed of fat, cholesterol, and other substances – accumulates on the inner walls of arteries, leading to restricted blood flow. The clinical presentation of PAD is diverse, ranging from being asymptomatic to exhibiting classic intermittent claudication, which involves pain, cramping, or fatigue in the legs during physical activity that is relieved by rest. In severe instances, PAD can progress to rest pain, tissue ulcers, gangrene, and may even necessitate amputation. Epidemiological data indicate a global prevalence of 1.52%^[3]^ for PAD, with a trend of gradual increase in recent years.

Emerging evidence suggests that several micronutrients – including minerals and vitamins^[4,5]^ – may play a protective role in the development of PAD. Among vitamins, both vitamin E and vitamin C, as well as supplementation with potassium, zinc, and magnesium, have demonstrated cardioprotective effects.^[4,6,7]^ These benefits are thought to arise from mechanisms such as the reduction of oxidative stress, stabilization of atherosclerotic plaques, and regulation of blood pressure fluctuations. For instance, low serum magnesium has been proposed as a potential risk factor for PAD,^[8,9]^ particularly in populations with chronic conditions such as chronic kidney disease, though definitive conclusions remain elusive. Additionally, folate and B vitamins (B2, B6, B12) are known to lower homocysteine levels, which have been implicated in increased vascular smooth muscle cell proliferation and collagen synthesis.^[10,11]^ Elevated homocysteine can also compromise vascular integrity by inducing endothelial dysfunction^[12]^ and reducing arterial wall elasticity.^[13]^ Despite these findings, most supporting evidence is derived from observational studies, and the potential for residual confounding remains. This underscores the need for research employing causal inference methods to clarify the true impact of micronutrients on PAD risk.

Mendelian randomization (MR) is a genetic epidemiology approach^[14,15]^ that leverages the random allocation of genetic variants at conception to assess causal relationships^[16]^ between exposures and health outcomes. By using genetic variants as instrumental variables (IVs), MR minimizes confounding and reverse causation, providing more robust evidence for causality than traditional observational studies. To address the unresolved causal links between micronutrient levels and PAD risk, we analyzed genome-wide association study (GWAS) summary statistics within a 2-sample MR framework. To provide supporting epidemiologic evidence in an independent population, we additionally analyzed National Health and Nutrition Examination Survey (NHANES) data, which offer a large, nationally representative sample, standardized ankle–brachial index (ABI) measurements for PAD ascertainment, and rich covariate data for confounder control.

## 2. Materials and methods

**Figure 1.**
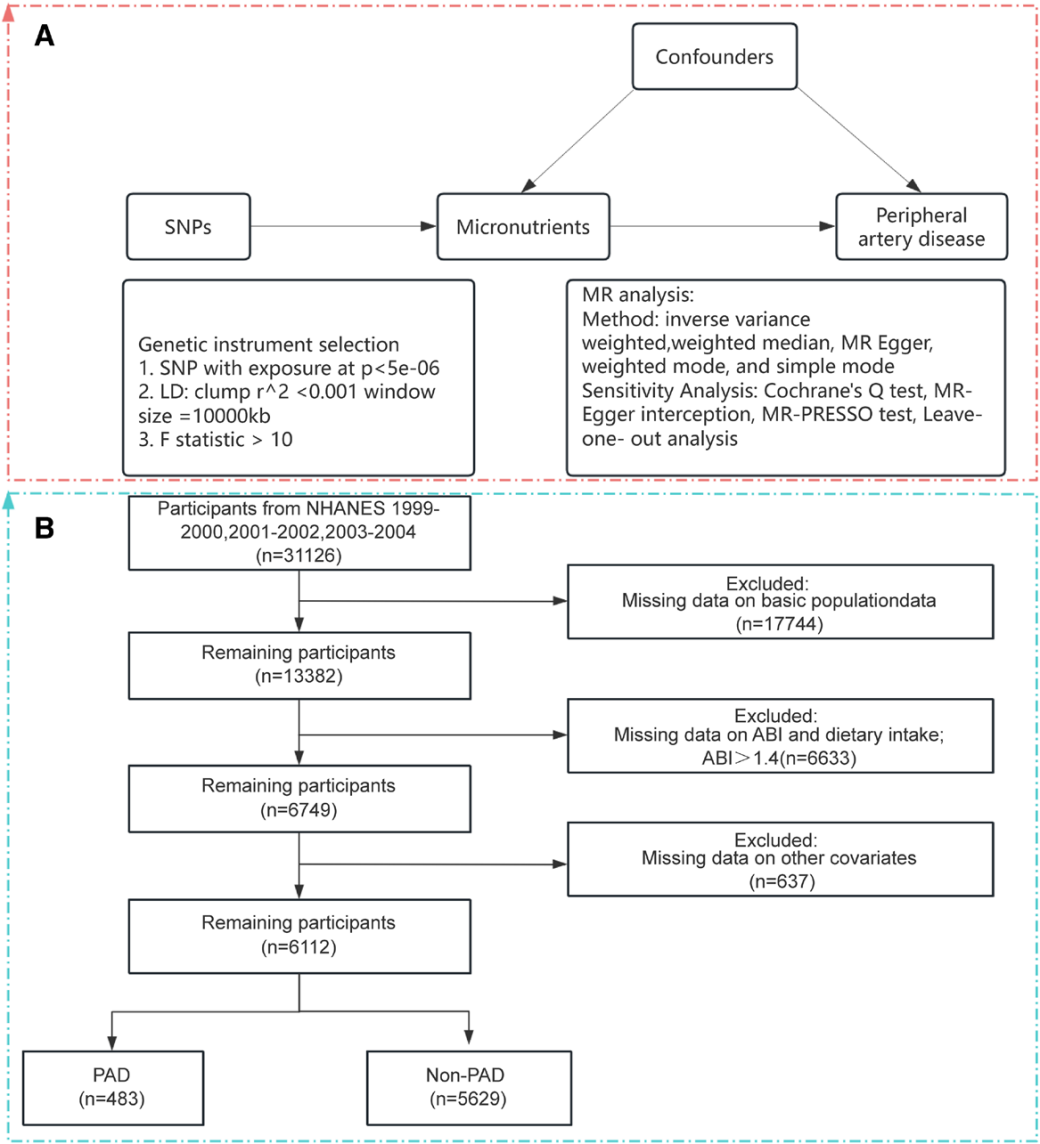
Participant selection flowchart: (A) Two-sample MR approach using SNPs as instrumental variables to assess the causal effect of micronutrients on peripheral artery disease (PAD); (B) Relationship between vitamin C intake and PAD. MR = Mendelian randomization, NHANES = National Health and Nutrition Examination Survey, PAD = peripheral artery disease, SNP = single-nucleotide polymorphism.

This study was conducted in 2 phases. First, we performed a 2-sample MR analysis using summary statistics for micronutrients and PAD from GWAS to establish genetic evidence for causal relationships (Fig. 1A). Second, we analyzed the association between dietary intake of candidate micronutrients and PAD risk using data from NHANES, adjusting for potential confounders (Fig. 1B).

### 2.1. MR analysis

#### 2.1.1. Study design

We applied a 2-sample MR framework using single-nucleotide polymorphisms (SNPs) as IVs to infer causality between circulating micronutrients and PAD risk. The 3 core MR assumptions were upheld: relevance: SNPs are strongly associated with each exposure; independence: SNPs are uncorrelated with confounders; exclusion restriction: SNPs affect PAD only via the exposure. A reverse MR approach was also implemented to adjust for correlated micronutrients.

#### 2.1.2. Data source

Due to the absence of suitable GWAS data for Chinese or other Asian‐ancestry populations, we identified summary statistics for 15 circulating micronutrients from European‐ancestry GWAS in the GWAS Catalog: copper,^[17]^ calcium, carotene, folate, iron, magnesium, potassium, selenium,^[17]^ zinc,^[17]^ and vitamins A, B6, B12, C, D, and E. GWAS data for thiamin (vitamin B1), riboflavin (B2), and vitamin D3 were not available and were therefore excluded. These studies included participants of European descent recruited from the USA, the Netherlands, Australia (TwinsUK), Germany, and Canada, minimizing sample overlap with the PAD outcome dataset. Cohort characteristics, GWAS IDs, and consortia are summarized in Table 1.

**Table 1:** Details of genome-wide association studies for micronutrient exposures used in Mendelian randomization analyses.

Traits	GWAS ID	Consortium	Ancestry
Copper	ieu-a-1073	NA	European
Calcium	ukb-b-8951	MRC-IEU	
Carotene	ukb-b-16202	MRC-IEU	
Folate	ukb-b-11349	MRC-IEU	
Iron	ukb-b-20447	MRC-IEU	
Magnesium	ukb-b-7372	MRC-IEU	
Potassium	ukb-b-17881	MRC-IEU	
Selenium	ieu-a-1077	NA	
Vitamin A	ukb-b-9596	MRC-IEU	
Vitamin B12	ukb-b-19524	MRC-IEU	
Vitamin B6	ukb-b-7864	MRC-IEU	
Vitamin C	ukb-b-19390	MRC-IEU	
Vitamin D	ukb-b-18593	MRC-IEU	
Vitamin E	ukb-b-6888	MRC-IEU	
Zinc	ieu-a-1079	NA	

GWAS = genome-wide association study.

PAD summary statistics were obtained from the GWAS conducted by Sakaue et al^[18]^ (2021; GWAS ID: ebi-a-GCST90018890), which comprised 7114 PAD cases and 475,964 controls and analyzed approximately 24 million SNPs.

All contributing studies received ethical approval, and all participants provided informed consent.

#### 2.1.3. Instrumental variable selection

We applied uniform selection criteria to identify genetic variants for 15 micronutrients. Given the scarcity of SNPs reaching genome-wide significance for these metabolites, we adopted a more lenient threshold^[19]^ of *P* < 5 × 10^−6^. Significant SNPs were then pruned by linkage disequilibrium^[20]^ (*r*^2^ < 0.001, window = 10,000 kb) to ensure independence. Finally, to guard against weak instrument bias, we computed the *F*-statistic for each SNP and excluded those with *F* < 10.^[21]^

#### 2.1.4. MR analysis

The inverse-variance-weighted (IVW) method^[22]^ was applied as the primary estimator of causal effects between circulating micronutrients and PAD (*P* <.05). IVW combines SNP-specific Wald ratios weighted by the inverse of their variances, assuming valid instruments, but may remain susceptible to unmeasured confounding and pleiotropy.

Complementary MR methods (weighted median,^[23]^ MR-Egger,^[24]^ simple mode,^[25]^ and weighted mode^[26]^) were used to evaluate result consistency. Heterogeneity was tested using Cochran Q^[27]^ (*P* <.05 and *I*^2^ >25% indicating heterogeneity). Leave-one-out analysis identified any single SNP driving the association. Horizontal pleiotropy was assessed via the MR-Egger intercept^[24]^ (*P* > .05 denotes no pleiotropy) and the MR-PRESSO global test. All MR analyses were conducted in R (v4.4.3) with the TwoSampleMR package (v0.6.17).

Micronutrients were considered candidate causal factors if they: showed consistent associations across all 5 MR methods; demonstrated no evidence of horizontal pleiotropy; and lacked influential SNPs in leave-one-out analysis.

#### 2.1.5. Reverse MR analysis

For our study, we extracted SNPs associated with PAD from GWAS summary statistics using a stringent significance threshold (*P*-value <5 × 10^−6^). We then chose SNPs that were independent (with an r-squared value <0.001) within the European panel. These independent SNPs were utilized as IVs in MR analysis to investigate a potential candidate micronutrient.

Summary statistics for the exposures were obtained from large-scale GWAS datasets. Outcome summary statistics for PAD were extracted from large-scale GWAS datasets (GWAS ID: ebi-a-GCST90018890). SNPs associated with each exposure were selected using a genome-wide significance threshold of *P* <5 × 10^−6^, with clumping parameters set to 10,000 kb and *r*^2^ <0.001 to ensure independence. The exposure and outcome datasets were harmonized to align effect alleles and ensure consistency.

### 2.2. Overview of NHANES

#### 2.2.1. Study design and population

Our analyses utilized data from the NHANES, overseen by the National Center for Health Statistics. All study protocols received approval from the National Center for Health Statistics Research Ethics Review Board, and informed written consent was obtained from each participant.

This study utilized data from 3 NHANES survey cycles conducted between 1999 and 2004 (total n = 31,126). Each cycle comprised 5 components: demographics, dietary intake, examination results, laboratory findings, and questionnaire responses. Participants aged 40 years or older with a valid ankle–brachial index (ABI ≤1.4) and complete dietary intake records were identified (n = 6749). After excluding individuals missing covariate data (n = 637), the final analytic sample consisted of 6112 participants (Fig. 1B).

#### 2.2.2. Exposure and outcome variates

NHANES dietary intake data were used to estimate the types and amounts of foods and beverages consumed during the 24 hours prior to the interview. This included energy, nutrients, and other food components. After the dietary recall, participants answered questions about water consumption, salt use, and whether their intake was typical for them. All interviewers completed standardized training and supervised practice to ensure the accuracy of the 24-hour dietary recalls. Individual thiamin intake was calculated from these records.

ABI measurements were performed in participants over 40 years old. Systolic blood pressure was measured in the right arm (or left if necessary) and both ankles after a brief rest. For those aged 40 to 59, 2 measurements were taken; for those 60 and older, 1 measurement was taken. ABI was calculated as the ratio of ankle to arm systolic blood pressure. PAD was defined as an ABI <0.9.

#### 2.2.3. Covariates

Standardized questionnaires were used to collect data on age, sex, race/ethnicity, education, family income, smoking status, and medical history (including hypertension, coronary heart disease (CHD), diabetes, and tumor). Body mass index (BMI, kg/m^2^) was measured at the Mobile Examination Center. Detailed variable definitions and measurement methods are available on the official NHANES website. Race/ethnicity was categorized as Mexican American, other Hispanic, non-Hispanic White, non-Hispanic Black, or other. Education was grouped into less than high school, high school or equivalent, and college or above. Family income was classified as low (poverty income ratio (PIR) <1.30) or high (PIR ≥1.30). Smoking status was defined as current, former, or never smoker.

#### 2.2.4. Statistical analysis

Participant characteristics were compared across quartiles of dietary intake of the potential candidate micronutrient. Categorical variables were summarized as proportions and compared using chi-square tests, while continuous variables were presented as means ± SD and compared using ANOVA. The association between dietary intake of potential candidate micronutrients and PAD risk was evaluated using binary logistic regression, with covariates selected based on clinical relevance and prior studies. Three models were constructed: Model 1 (unadjusted), Model 2 (adjusted for age, race, sex, and PIR), and Model 3 (fully adjusted for all covariates).

Restricted cubic spline models were used to assess nonlinear dose–response relationships between dietary intake of potential candidate micronutrients and PAD risk, with intake modeled as a continuous variable and knots at the 5th, 35th, 65th, and 95th percentiles. nonlinearity was tested by adding a quadratic term; if present, a 2-piecewise regression was applied to identify threshold effects. Subgroup and sensitivity analyses were performed to test robustness. We screened dietary micronutrient intakes and other continuous variables for outliers and implausible values. When necessary, sensitivity analyses were performed to evaluate the robustness of the results to extreme observations. All analyses were conducted in R (v4.4.3), with *P* <.05 considered statistically significant.

## 3. Results

### 3.1. Mendelian analysis of micronutrients intake and PAD

**Figure 2.**
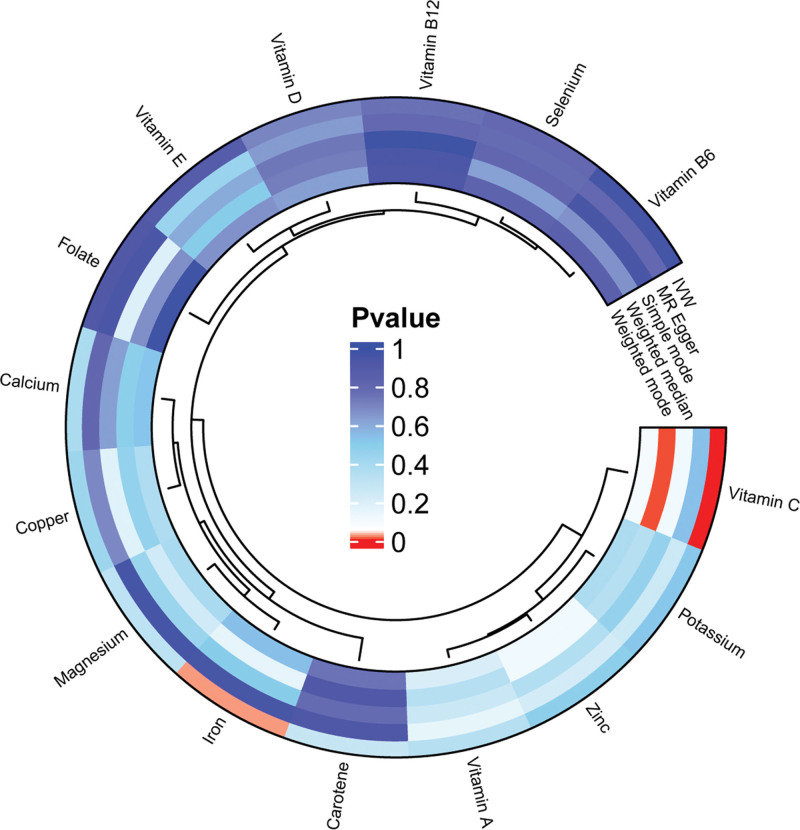
Screening overview of MR results for 15 circulating micronutrients and peripheral artery disease. MR = Mendelian randomization.

**Figure 3.**
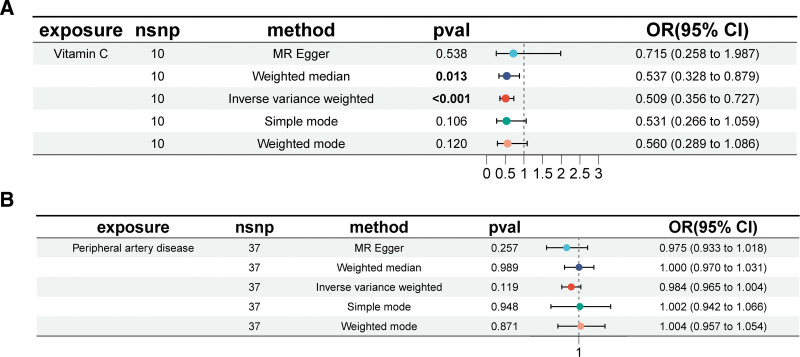
Forest plot illustrating the causal effects: (A) the impact of vitamin C on the risk of PAD; (B) the effect of peripheral artery disease on vitamin C levels. CI = confidence interval, OR = odds ratio, PAD = peripheral artery disease.

We performed a comprehensive MR analysis to evaluate the causal effects of 15 circulating micronutrients on PAD. After rigorous instrument selection, a total of 188 SNPs (see Table S1, Supplemental Digital Content, https://links.lww.com/MD/R596) were included as genetic instruments. A circular plot was used to visualize the micronutrients under investigation (Fig. 2). Although 15 micronutrients were initially screened, only vitamin C consistently demonstrated a significant association with PAD across all 5 MR methods (IVW, MR-Egger, weighted median, simple mode, weighted mode), with a significance threshold of *P* <.05 in the IVW analysis. Heterogeneity was considered negligible when the corresponding test yielded a *P*-value >.05. Ultimately, higher genetically predicted vitamin C levels were associated with a lower risk of PAD (OR = 0.509, 95% confidence interval [CI]: 0.356–0.727; *P* <.001) (Fig. 3A).

**Figure 4.**
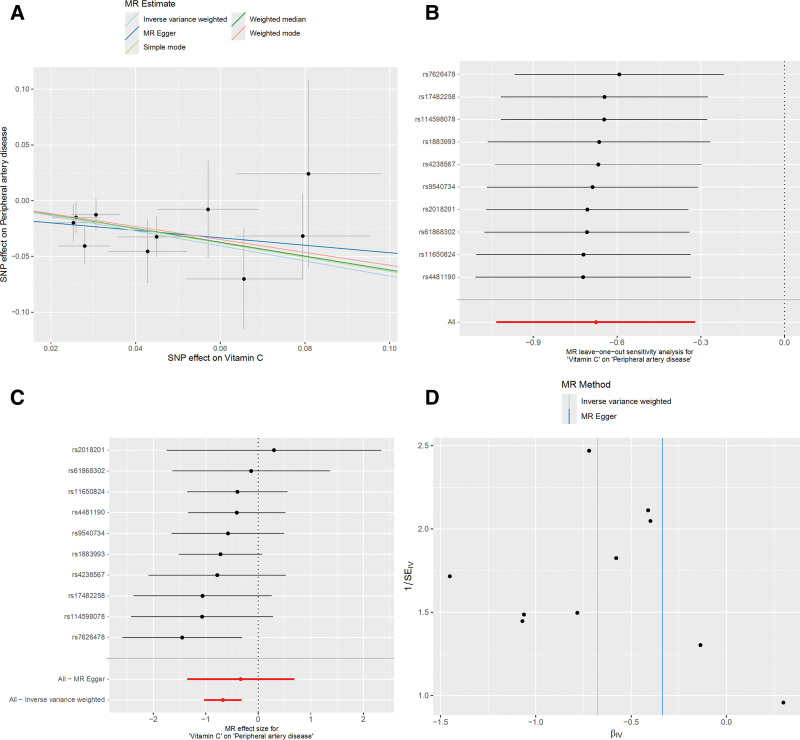
MR plots for the relationship of vitamin C with peripheral artery disease. (A) Scatter plot of SNP effects. (B) Forest plot presenting effect sizes estimated by individual and combined SNP MR analyses. (C) Leave-one-out plot. (D) Funnel plot. MR = Mendelian randomization, SNP = single-nucleotide polymorphism.

To ensure the robustness of our findings and account for potential confounding, we applied a suite of sensitivity analyses, including MR-Egger regression, weighted median, simple mode, and weighted mode, in addition to the standard IVW approach (Fig. 4). Pleiotropy and heterogeneity were further assessed (Tables S2 and 3, Supplemental Digital Content, https://links.lww.com/MD/R597). The *F*-statistics indicated no evidence of weak instrument bias, and MR-PRESSO did not detect any outliers. These rigorous approaches minimized potential biases and enhanced the reliability of our results.

In the context of reverse causation, where genetic predisposition to PAD is considered as an exposure, we conducted MR analyses to investigate the causal impact of PAD on vitamin C. Across all MR methodologies employed, no indication of a causal association between PAD and vitamin C was observed (IVW odds ratio [OR] = 0.984, 95% CI, 0.965–1.004; *P* = .119, Fig. 3B).

### 3.2. Association between dietary vitamin C intake and PAD from NHANES

#### 3.2.1. Study population and characteristics

A total of 6112 participants aged 40 years or older were included in the analysis, with an overall PAD prevalence of 7.9%. Table 2 shows baseline characteristics of participants, stratified by quartiles of dietary vitamin C intake (<27.47, 27.47–64.66, 64.66–128.93, ≥128.93 mg/day). Significant differences were observed among quartile groups in age, PIR, education, literacy, hypertension, smoking, and diabetes mellitus (all *P* <.05), while sex, BMI, tumor, and CHD distributions were similar (all *P* >.05). Participants with higher vitamin C intake tended to be younger, more likely to be male, never smokers, and had higher education and PIR. Notably, higher vitamin C intake was associated with a lower PAD rate (Quartile 1: 9.1%, Quartile 2: 7.9%, Quartile 3: 7.9%, Quartile 4: 6.7%, *P* = .12).

**Table 2:** Baseline characteristics of the participants stratified by the dietary vitamin C intake quartiles.

Characteristic	Overall, N = 6,112*	Q1 (<27.47)*	Q2 (27.47–64.66)*	Q3 (64.66–128.93)*	Q4 (>128.93)*	*P*-value†
Age (year)	60 (13)	57 (13)	59 (13)	63 (13)	60 (13)	<.001
Sex, male	3116 (51%)	761 (50%)	765 (50%)	766 (50%)	824 (54%)	.069
Family poverty income ratio	2.48 (1.61)	2.00 (1.56)	2.67 (1.62)	2.58 (1.59)	2.73 (1.62)	<.001
BMI, kg/m^2^	27.8 (5.6)	27.7 (5.7)	28.0 (5.8)	27.8 (5.2)	27.7 (5.7)	.086
Age group						
40–59 yr	2960 (48%)	822 (54%)	767 (50%)	637 (42%)	734 (48%)	<.001
60–79 yr	2559 (42%)	598 (39%)	634 (41%)	686 (45%)	641 (42%)	
80 + yr	593 (9.7%)	108 (7.1%)	127 (8.3%)	205 (13%)	153 (10%)	
Race						
Non-Hispanic White	1271 (21%)	312 (20%)	311 (20%)	312 (20%)	336 (22%)	<.001
Non-Hispanic Black	1098 (18%)	326 (21%)	244 (16%)	261 (17%)	267 (17%)	
Mexican American	3364 (55%)	814 (53%)	878 (57%)	863 (56%)	809 (53%)	
Other/multiracial	230 (3.8%)	55 (3.6%)	63 (4.1%)	51 (3.3%)	61 (4.0%)	
Other Hispanic	149 (2.4%)	21 (1.4%)	32 (2.1%)	41 (2.7%)	55 (3.6%)	
Education level						
Less than high school	1464 (24%)	370 (24%)	358 (23%)	401 (26%)	335 (22%)	<.001
High School	1984 (32%)	611 (40%)	485 (32%)	461 (30%)	427 (28%)	
More than high school	2664 (44%)	547 (36%)	685 (45%)	666 (44%)	766 (50%)	
Income						
low income	4546 (74%)	1039 (68%)	1139 (75%)	1177 (77%)	1191 (78%)	<.001
high income	1566 (26%)	489 (32%)	389 (25%)	351 (23%)	337 (22%)	
Peripheral artery disease	483 (7.9%)	139 (9.1%)	121 (7.9%)	120 (7.9%)	103 (6.7%)	.12
Smoke						
Current smoker	1181 (19%)	449 (29%)	299 (20%)	216 (14%)	217 (14%)	<.001
Former smoker	2112 (35%)	495 (32%)	523 (34%)	570 (37%)	524 (34%)	
Never smoker	2819 (46%)	584 (38%)	706 (46%)	742 (49%)	787 (52%)	
BMI group						
Normal (18.5 to < 25)	1599 (26%)	414 (27%)	361 (24%)	413 (27%)	411 (27%)	.032
Obese (30 or greater)	2027 (33%)	523 (34%)	541 (35%)	476 (31%)	487 (32%)	
Overweight (25 to < 30)	2427 (40%)	572 (37%)	616 (40%)	628 (41%)	611 (40%)	
Underweight (<18.5)	59 (1.0%)	19 (1.2%)	10 (0.7%)	11 (0.7%)	19 (1.2%)	
Coronary heart disease	402 (6.6%)	92 (6.0%)	107 (7.0%)	104 (6.8%)	99 (6.5%)	.7
Hypertension	2608 (43%)	642 (42%)	691 (45%)	672 (44%)	603 (39%)	.008
Tumor	764 (13%)	191 (13%)	174 (11%)	220 (14%)	179 (12%)	.054
Diabetes	864 (14%)	210 (14%)	203 (13%)	250 (16%)	201 (13%)	.036

* Median (SD); n (%).

† Kruskal–Wallis rank sum test; Pearson Chi-squared test.

#### 3.2.2. Association of dietary vitamin C intake with PAD

Table 3 presents the results of univariate and multivariable logistic regression analyses. Three logistic regression models were constructed to assess the independent effect of vitamin C intake on PAD risk. When vitamin C intake was categorized by quartiles (first quartile as reference), participants in the second to fourth quartiles had a lower risk of PAD in all models. Univariate ORs (95% CI) for PAD from lowest to highest intake were 1.00 (reference), 0.86 (0.67–1.11), 0.85 (0.66–1.10), and 0.72 (0.55–0.94). After multivariate adjustment for age, sex, race, education, PIR, BMI, smoking, and hypertension (Model 3), the adjusted ORs (95% CI) were 1.00 (reference), 0.89 (0.68–1.17), 0.74 (0.56–0.98), and 0.80 (0.60–1.06).

**Table 3:** Odds ratios and 95% confidence interval of the vitamin C quartiles for peripheral artery disease.

Characteristic	Model 1			Model 2			Model 3		
	OR	95% CI	*P*-value	OR	95% CI	*P*-value	OR	95% CI	*P*-value
Quantiles									
Q1	Ref	–	–	Ref	–	–	Ref	–	–
Q2	0.86	0.67–1.11	.2	0.84	0.64–1.10	.2	0.89	0.68–1.17	.4
Q3	0.85	0.66–1.10	.2	0.68	0.52–0.89	.006	0.74	0.56–0.98	.035
Q4	0.72	0.55–0.94	.016	0.71	0.53–0.94	.016	0.80	0.60–1.06	.12

Model 1 adjusted for none. Model 2 adjusted for age, sex, race, and PIR. Model 3 adjusted for all covariates.

CI = confidence interval, OR = odds ratio.

#### 3.2.3. Detection of nonlinear relationships

**Figure 5.**
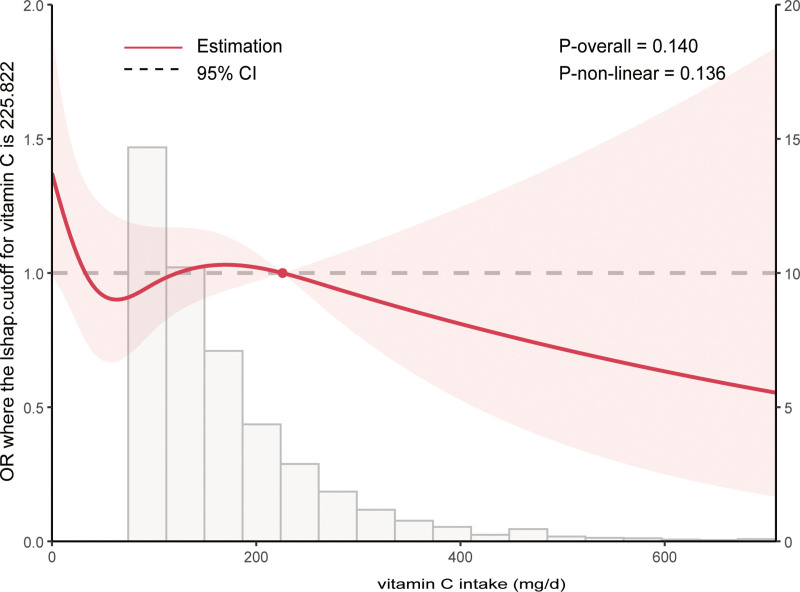
The restricted spline curve shows the relationship between dietary vitamin C intake and peripheral artery disease.

Multivariable-adjusted restricted cubic spline analysis (Fig. 5) showed an L-shaped association between vitamin C intake and PAD risk (p for nonlinearity = 0.136), with risk decreasing up to 225.82 mg/day and then stabilizing. In quartile analysis (Table 4), the second quartile (64.66–225.82 mg/day) was associated with a significantly lower PAD risk (adjusted OR = 0.79; 95% CI: 0.64–0.98), while the third quartile (≥225.82 mg/day) was not statistically significant (adjusted OR = 0.94; 95% CI: 0.63–1.38).

**Table 4:** Odds ratios and 95% confidence interval of vitamin C categories for peripheral artery disease.

Characteristic	Crude model			Adjusted model		
	OR	95% CI^1^	*P*-value	OR	95% CI	*P*-value
Categories						
Q1 (<64.66)	Ref	–	–	Ref	–	–
Q2 (64.66–225.82)	0.86	0.71–1.05	.13	0.79	0.64–0.98	.030
Q3 (≥225.82)	0.78	0.53–1.11	.2	0.94	0.63–1.38	.8
Threshold						
Q1 (<225.82)	Ref	–	–	Ref	–	–
Q2 (≥225.82)	0.83	0.57–1.17	.3	1.05	0.71–1.52	.8

Crude model adjusted for none. Adjusted model adjusted for all covariates.

CI = confidence interval, OR = odds ratio.

To further assess the stability of the dose–response relationship, we performed sensitivity analyses using different numbers of knots in the restricted cubic spline models. The main analysis with 4 knots produced a smoother curve and a threshold at 225.82 mg/day (*P* for nonlinearity = 0.136). Sensitivity analysis with 5 knots identified a threshold at 216.0 mg/day and a borderline significant nonlinearity (*P* = .056), but the curve showed more fluctuation. Therefore, the 4-knot model was chosen for primary interpretation, and the 5-knot results support the robustness of the findings.

#### 3.2.4. Stratification analysis

**Figure 6.**
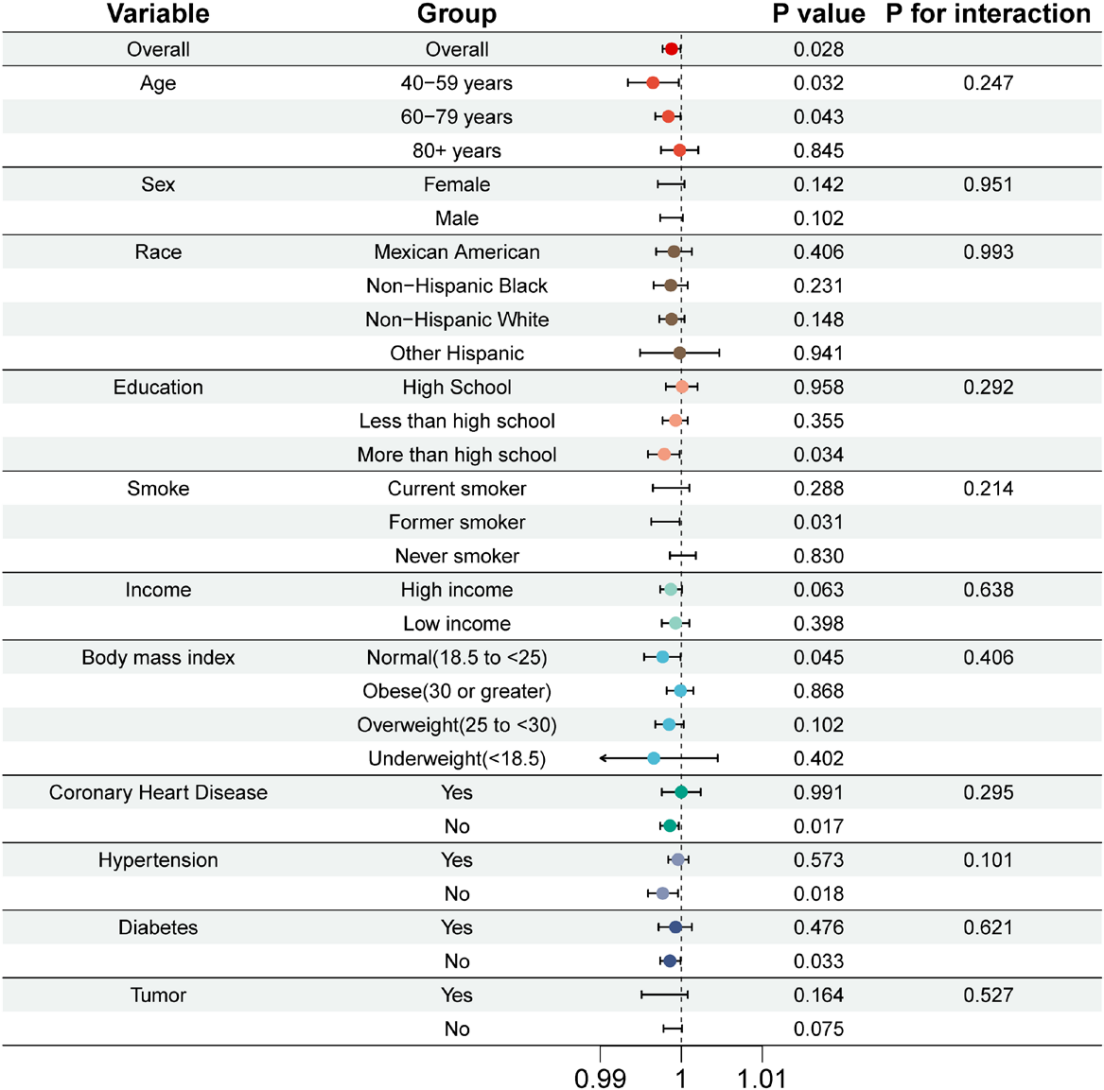
Subgroup analysis of the association between vitamin C intake and risk of peripheral artery disease. The results were adjusted for all covariates except the corresponding stratification variable. CI = confidence interval, HR = hazard ratio.

Stratified analyses evaluated the association between dietary vitamin C intake and PAD risk across subgroups by age, sex, income, education, smoking status, and disease conditions (hypertension, diabetes, CHD, tumor) (Fig. 6). Vitamin C intake was treated as a continuous variable. Significant negative associations were observed in participants under 80 years, those with education above high school, normal BMI, former smokers, and those without CHD, hypertension, or diabetes.

## 4. Discussion and conclusions

This study comprehensively examined the relationship between micronutrient status and PAD using both genetic and observational approaches. First, we employed a 2-sample MR analysis to investigate the causal relationships between micronutrients and PAD risk. Our principal finding demonstrates that genetically predicted elevated plasma vitamin C levels are causally associated with reduced PAD risk. We then validated these findings in a large population-based sample from NHANES, demonstrating that increased dietary vitamin C intake is associated with a lower risk of PAD, especially in certain subgroups. Together, our results suggest a protective role of vitamin C in PAD, though the effect size and statistical significance vary across populations and intake levels.

Our study provides strong genetic and observational evidence that vitamin C plays a protective role in PAD. MR analysis revealed that higher genetically predicted vitamin C levels were significantly associated with a reduced risk of PAD (IVW OR = 0.509, 95% CI: 0.356–0.727; *P* <.001), and no evidence of reverse causality was found. These findings were validated in the NHANES cohort, where increased dietary vitamin C intake was independently linked to lower PAD risk, with an L-shaped dose–response relationship and a threshold at 225.82 mg/day. Subgroup analyses showed the protective effect was consistent across various populations, especially in those under 80 years, with higher education, normal BMI, and without major comorbidities. Recent studies have similarly combined MR with NHANES analyses to triangulate genetic and population-based evidence for micronutrients/antioxidants and cardiometabolic outcomes, supporting the feasibility and interpretability of this dual-evidence framework.^[28,29]^

Observational studies have long suggested close associations between micronutrients and PAD. For instance, analyses of NHANES data revealed that vitamins A, C, B6, E, and folate were associated with reduced PAD risk.^[30]^ Additionally, hypomagnesemia^[9]^ and vitamin D^[31]^ have been linked to PAD risk. However, these observational studies, due to their inherent limitations – namely, the inability to completely eliminate confounding factors (such as other vitamins and minerals) and reverse causation – cannot establish definitive causal relationships. Our MR study precisely fills this evidence gap. By utilizing genetic variants associated with micronutrient levels as IVs, MR analysis simulates a “natural randomized trial,” substantially reducing confounding and reverse causation biases inherent in traditional epidemiological studies. The findings of this study provide causal explanations for the associations observed in observational studies and powerfully demonstrate that low vitamin C levels represent a pathogenic risk factor for PAD, rather than merely an accompanying biomarker.

Classical cohort studies^[32]^ have previously found that serum vitamin C levels in PAD patients are significantly lower than in healthy controls, and low vitamin C levels are associated with elevated inflammatory markers (such as C-reactive protein, CRP) and worse clinical presentations (such as shorter claudication distances). Furthermore, analysis^[33]^ of NHANES data revealed that in smoking populations – a group with high oxidative stress – low vitamin C levels are significantly associated with increased PAD risk.

Our study results are highly consistent with the well-established biological functions of vitamin C, which collectively explain its potential protective effects against atherosclerosis^[34,35]^ (the primary pathological basis of PAD):

**Potent antioxidant activity**: Vitamin C serves as a key water-soluble antioxidant in the body,^[36]^ effectively scavenging reactive oxygen species and inhibiting low-density lipoprotein oxidation^[37]^ and lipoprotein(a),^[34]^ thereby delaying the earliest stages of atherosclerotic plaque formation.

**Anti-inflammatory properties**: Our study results align with findings from observational studies showing negative correlations between vitamin C levels and CRP.^[32]^ Vitamin C may reduce chronic inflammatory responses in vessel walls by inhibiting inflammatory pathways such as nuclear factor κB.^[38]^

**Improvement of endothelial function**: Vitamin C is crucial for maintaining endothelial progenitor cell function^[39]^ and promoting nitric oxide bioavailability.^[40]^ It enhances NO production by regenerating tetrahydrobiopterin (BH4)-a key cofactor for endothelial nitric oxide synthase-thereby promoting vasodilation, inhibiting platelet aggregation, and preventing leukocyte adhesion.^[41]^

Importantly, our NHANES analysis revealed an L-shaped association between vitamin C intake and PAD risk, with a threshold effect at 225.82 mg/day. This suggests that increasing vitamin C intake up to a certain level may reduce PAD risk, but further increases may not confer additional benefit. The protective association was most evident in specific subgroups, highlighting the importance of personalized nutrition strategies.

We did not identify significant associations between other minerals or vitamins and PAD in MR analyses. This contrasts with some previous observational studies, which may be explained by confounding, limited sample size, or lack of statistical power in genetic analyses. The literature on micronutrient supplementation is limited to a few small-scale studies targeting specific patient subgroups, precluding specific recommendations. Our results suggest that, among the micronutrients studied, only vitamin C shows robust evidence for a causal role in PAD.

The identification of vitamin C as a protective factor against PAD has potential clinical implications. While randomized controlled trials are needed to definitively establish the efficacy of vitamin C supplementation in PAD prevention and treatment, our findings suggest that maintaining adequate vitamin C levels may be beneficial for cardiovascular health, particularly in populations at high risk for PAD. Healthcare providers may consider assessing vitamin C status in patients with PAD risk factors and recommend dietary modifications or supplementation when deficiencies are identified, but current evidence does not support universal supplementation.

The main strength of this study is the integration of MR analysis with independent clinical validation using NHANES data, which provides robust and complementary evidence for the association between vitamin C and PAD risk. By combining genetic and observational approaches, we minimized confounding, reverse causation, and population stratification biases. The use of multiple MR methods and comprehensive sensitivity analyses further ensured the reliability and validity of our findings. Additionally, our validation strategy meets the standards required for bioinformatics and generalizability of the results.

This study has several limitations. First, because only a limited number of variants reached conventional genome-wide significance for some micronutrients, we adopted a less stringent threshold (*P* <5 × 10^−6^) for instrument selection. Although we excluded variants with *F*-statistics <10 and performed extensive sensitivity analyses, using a relaxed threshold may still increase the risk of weaker instruments and potential bias. Second, the MR analysis was based on European-ancestry genome-wide association studies, whereas NHANES includes multiple racial/ethnic groups; although we adjusted for race/ethnicity in NHANES, differences in genetic architecture and exposure distributions may limit generalizability. Third, the NHANES component is cross-sectional; therefore, the observed associations reflect PAD risk in terms of the odds of prevalent PAD and cannot establish causality or fully exclude reverse causation. Fourth, some important confounding factors were not fully accounted for in the NHANES analyses, including dietary supplement use, physical activity, and chronic inflammatory conditions (for example, C-reactive protein), which may result in residual confounding. Finally, plasma/serum vitamin C was not used for validation because it was only available in limited NHANES cycle (2003–2004); restricting analyses to this subset would substantially reduce sample size and statistical power and may introduce selection bias.

In summary, our MR screening identified vitamin C as the only micronutrient showing consistent evidence for a protective association with PAD. The supportive NHANES analyses further suggested that higher dietary vitamin C intake was associated with lower odds of prevalent PAD, with an L-shaped dose–response pattern. Collectively, these findings indicate that vitamin C may play a protective role in PAD; however, stronger instruments, longitudinal cohorts, biomarker-based validation, and well-designed randomized controlled trials are needed to confirm causality and inform prevention and management strategies.

## Acknowledgments

We would like to thank all participants in this study.

## Author contributions

**Conceptualization:** Zhiyong Dong, Lisong Hao.

**Data curation:** Zhiyong Dong, Lisong Hao.

**Formal analysis:** Zhiyong Dong.

**Funding acquisition:** Zhiyong Dong.

**Investigation:** Zhiyong Dong, Qingyun Wang, Lisong Hao.

**Methodology:** Zhiyong Dong, Qingyun Wang.

**Project administration:** Zhiyong Dong.

**Resources:** Zhiyong Dong.

**Software:** Zhiyong Dong.

**Supervision:** Qingyun Wang, Lisong Hao.

**Validation:** Lisong Hao.

**Visualization:** Zhiyong Dong.

**Writing – original draft:** Zhiyong Dong, Lisong Hao.

**Writing – review & editing:** Qingyun Wang, Lisong Hao.

## Supplementary Material




