# Supplementary material for: Potential protective role of vitamin C in peripheral artery disease: A Mendelian randomization and NHANES analysis
Source: Medicine (Baltimore). 2026 Apr 3;105(14):e48220. doi: 10.1097/MD.0000000000048220 (PMC13052945; doi:10.1097/MD.0000000000048220)
Supplement: Supplementary file 2 [file medi-105-e48220-s002.pdf]

Table S2. Heterogeneity results from the Cochran's Q test of significant causal links between vitamin C and peripheral artery disease

exposure	outcome	method	Q	Q_df	Q_pval
Vitamin C	Peripheral artery disease	MR Egger	4.01	8	0.86
		Inverse variance weighted	4.50	9	0.87

Table S3. Pleiotropy results from Egger intercept analysis and MR presso between vitamin C and peripheral artery disease

**exposure**	**outcome**	**MR egger_intercept**	***P*val**	**MR-presso Global**
Vitamin C	Peripheral artery disease	-0.013	0.505	0.901
